# Identification of Mitochondrial DNA (NUMTs) in the Nuclear Genome of *Daphnia magna*

**DOI:** 10.3390/ijms21228725

**Published:** 2020-11-18

**Authors:** Krzysztof Kowal, Angelika Tkaczyk, Mariusz Pierzchała, Adam Bownik, Brygida Ślaska

**Affiliations:** 1 Faculty of Animal Sciences and Bioeconomy, Institute of Biological Bases of Animal Production, University of Life Sciences in Lublin, Akademicka 13 Str., 20-950 Lublin, Poland; krzysztof.kowal@up.lublin.pl (K.K.); angelika.tkaczyk@up.lublin.pl (A.T.); 2 Department of Genomics and Biodiversity, Institute of Genetics and Animal Biotechnology of the Polish Academy of Sciences, Postępu 36a Str., 05-552 Jastrzębiec, Poland; m.pierzchala@ighz.pl; 3 Faculty of Environmental Biology, Department of Hydrobiology and Protection of Ecosystems, University of Life Sciences in Lublin, Dobrzańskiego 37 Str., 20-262 Lublin, Poland; adam.bownik@up.lublin.pl

**Keywords:** mtDNA, NUMT, water flea, BLAST

## Abstract

This is the first study in which the *Daphnia magna* (*D. magna*) nuclear genome (nDNA) obtained from the GenBank database was analyzed for pseudogene sequences of mitochondrial origin. To date, there is no information about pseudogenes localized in *D. magna* genome. This study aimed to identify NUMTs, their length, homology, and location for potential use in evolutionary studies and to check whether their occurrence causes co-amplification during mitochondrial genome (mtDNA) analyses. Bioinformatic analysis showed 1909 fragments of the mtDNA of *D. magna*, of which 1630 were located in ten linkage groups (LG) of the nDNA. The best-matched NUMTs covering >90% of the gene sequence have been identified for two mt-tRNA genes, and they may be functional nuclear RNA molecules. Isolating the total DNA in mtDNA studies, co-amplification of nDNA fragments is unlikely in the case of amplification of the whole tRNA genes as well as fragments of other genes. It was observed that *TRNA-MET* fragments had the highest level of sequence homology, thus they could be evolutionarily the youngest. The lowest homology was found in the D-loop-derived pseudogene. It may probably be the oldest NUMT incorporated into the nDNA; however, further analysis is necessary.

## 1. Introduction

*Daphnia*, commonly known as the water flea, are small crustaceans usually inhabiting freshwater ponds and lakes on all continents of the globe. It has long been used as a model for the elucidation of animal responses and adaptations to environmental changes [1]. It has been also used in diverse biological research areas such as ecology, ecotoxicology, evolution, and reproductive biology due to its important position in the aquatic food chain, a high degree of phenotypic plasticity, and cyclical parthenogenesis responding to environmental stimuli [1,2,3,4,5,6,7]. Its sensitive behavioral and physiological responses are parameters used as biomarkers of the effect induced by various substances [1,2,3,4,5,6,7,8]. Thus, it has been used for reproduction tests, acute toxicity studies, and chronic toxicity tests in the OECD Guidelines [9,10]. The current state of knowledge of the nuclear genome of *Daphnia magna* is based on reports by Routtu et al. [11,12], Dukić et al. [13], and Lee et al. [14]. Low- and high-density genetic linkage maps were obtained, in which they assembled the whole genome sequence of *D. magna*. Specific genetic markers from a high-resolution genetic linkage map of *D. magna* Xinb3 were evaluated in toxicological studies by Korea Institute of Toxicology (KIT) [14].

Genomic resources are steadily being developed for many species of the genus Daphnia. In particular, a database of around 12,000 expressed sequence tags (EST) is currently available (http://wfleabase.org) [15], providing a useful resource to isolate polymorphic genetic markers in this species. However, there is no information about pseudogenic sequences of mitochondrial origin (NUMTs) in the *D. magna* genome.

Nuclear DNA sequences that are homologous to the mitochondrial genome are often referred to as mitochondrial pseudogenes, or NUMTs [16]. NUMTs may differ in length and be as large as the full length of the mitochondrial genome [17]. It has been reported that the NUMT length is positively correlated with the genome size, suggesting potential roles of non-coding DNA gain and loss in NUMT accumulation [18]. NUMTs have been documented in almost all eukaryotic genomes studied [19]. The transfer of mitochondrial DNA sequences into the nuclear genome is an ongoing evolutionary process [20], which has markedly influenced the evolution and function of eukaryotic genomes [19]. Thus, NUMTs are good materials for studying the evolution of nuclear sequences without selective constraints [21]. However, because of their homology, NUMTs may confound mtDNA studies, as the NUMT co-amplification product could interfere with sequence analysis [22].

This is the first study in which the *D. magna* nuclear genome deposited in the GenBank database was analyzed for pseudogene sequences of mitochondrial origin. The present study aimed to identify NUMTs, their length, homology, and location for potential use in evolutionary studies and to check whether their occurrence causes co-amplification during mitochondrial genome analyses.

## 2. Results

Bioinformatic analysis showed 1909 fragments of the mitochondrial genome, of which 1630 fragments were located in ten linkage groups of the nuclear genome of *D. magna*. The other fragments were localized in scaffolds which were used during genome sequencing. All the NUMTs found in this research are listed in supplementary materials (Table S1). The total length of the NUMT sequences in the linkage groups corresponded to the number of fragments on individual LG (Table 1). The total length of NUMTs in the *D. magna* genome was 44.391 base pairs (bp), which accounted for 0.042% of the length of the nuclear genome. Their percentage content was 0.037% in the longest linkage group 2, in which 228 NUMTs were identified, and 0.047% in the shortest linkage group 10 (Table 1). The most frequently occurring fragments of the mtDNA sequence in the nuclear genome included *ND2* (115), *ND3* (113), *TRNA-CYS* (110), and 16S rRNA (105). However, the highest number of NUMTs was observed for the non-coding area, i.e., the D-loop (147) (Table 2). The lowest numbers of mtDNA fragments found in nDNA were observed mainly for genes encoding tRNA molecules: *TRNA-PRO* and *TRNA-MET* (6), *TRNA-TYR* and *TRNA-ASN* (4), and *TRNA-SER1* (2). The highest numbers of mtDNA fragments were recorded on LG2-228, and the lowest—on LG8-134 (Table 2).

The longest fragments of the mitochondrial genome present in the nuclear genome were observed for the D-loop (182 bp), *ND4* (108 bp), *ND3* (99 bp), and *ND5* and *COX3* (94 bp each) (Table 3). The 182-bp fragment of the D-loop constituted 63% of the entire sequence of this region. In contrast, in the case of the other protein-encoding genes, the fragment size ranged from 4% (*CYTB)* to 32% (*ATP8*). In turn, NUMTs were recorded among genes encoding tRNA, constituting over 90% of the mtDNA gene sequence: *TRNA-ARG* and *TRNA-THR* (95% each) and *TRNA-GLU* (97%). All the analyzed sequence fragments had a minimum length in the range of 16–25 bp (Table 3).

Of the 1630 NUMTs (Table 1), 253 fragments, representing 16% of all NUMTs, showed 100% homology with the mtDNA gene sequences. 100% sequence homology for *TRNA-MET* was found for all 6 NUMTs (Table 2 and Table 4). 23 NUMTs whose sequence homology was 100% were observed for the D-loop region and 21 NUMTs for the gene *ND3*. In contrast, in the case of genes *TRNA-SER1* and *TRNA-ALA*, no NUMTs with 100% sequence homology were observed (Table 2). The mean values of the percentage of sequence identity ranged from 88.0% (*ATP8)* to 100% (*TRNA-MET*). The percentage identity for the individual linkage groups was in the range of 90–91%. The largest homology was recorded on LG10 (90.7%) and the lowest—on LG6 (90.2%) (Table 4). At least one sequence with 100% homology was identified on each of the linkage groups.

Table 5 compares the NUMTs’ number and length with mtDNA fragments found in pseudogenes and transcriptome of *D. magna*. The overall number of mtDNA fragments in pseudogenes was 599 and their highest length was estimated between 18 bp (*TRNA-MET*) to 99 bp (*ND3*). The lowest number of mtDNA fragments being part of pseudogenes was observed for *TRNA-PRO* (1), *ATP8* (2), and *TRNA-ASP* and *TRNA-ILE* (3). The highest number of these fragments was observed for *16s rRNA* (63) and D-loop (48). In the case of mtDNA fragments found in the transcriptome, the highest number was observed for *TRNA-CYS* (101) and *16S rRNA* (100). The lowest number of mtDNA fragments was found for *TRNA-SER1* and *TRNA-PHE* gene (1). The highest length ranged between 20 bp to 64 bp. The main localization of mtDNA fragments in transcriptome was in different mRNA transcript variants of various protein-coding genes as well as ncRNA and misc_RNA sequences (Table S1). The overall number of mtDNA fragments in transcriptome was 1275.

## 3. Discussion

This is the first study in which the *D. magna* nuclear genome deposited in the GenBank database was analyzed for pseudogene sequences of mitochondrial origin. The first complete information about the genome of *D. magna* was published by Lee et al. in 2019 [14]. To date, there is no information about pseudogenes localized in the genome of the water flea. Our research provides comprehensive bioinformatic details about the location of NUMTs found in the reference genome, which may be useful for future phylogenetic, evolution, and/or population analyses.

A computer-based search for NUMTs in the nuclear genome of 85 species of animals, plants, fungi, and protists showed that the total length of detected NUMTs varied from 0 to 823.9 kb per nuclear genome [23]. For instance, the NUMT content was 0 in *Anopheles gambiae*, 263.478 bp in *Homo sapiens*, and more than 800 kbp in *Oryza sativa* [23]. In the case of *D. magna* (Table 1), the overall length of NUMT in the nuclear genome was 44.391 bp. The total length of 24 NUMTs was 9.989 bp in the *Pteromalus puparum* genome, and 42.972 bp in *Nasoni vitripennis* [24], and more than 230 kbp in *Apis mellifera* [25]. There seems to be a positive correlation between the haploid genome sizes (C-values) and NUMT amount/prevalence in eukaryotes [26].

Another explanation for the differences in the total length of NUMTs is the fact that they accumulate in the genomes in a continuous evolutionary process [18]. Like in other species, e.g., *Myotis lucifugus* [27], the percentage of NUMTs in the genome was less than 0.1%. In contrast, the number and length of NUMTs may vary depending on the computer-based query for NUMTs in the BLAST tool, as in the case of the genome of *Canis lupus familiaris* [22,28]. In our study, the sequence search in BLASTN 2.6.0 implemented in CLC Genomics Workbench 12.0 yielded 1909 results, although 279 results were found in various scaffolds used during the sequencing of the *D. magna* genome. In this paper, however, the NUMT results from the scaffold were excluded due to the potential occurrence of artifacts created during sequencing, as observed by Shi et al. [27], where surprisingly, an entire mitochondrial genome was found in the scaffold AAPE02072785 in the *M. lucigufus* genome. It is also worth considering that, in this work, the BLAST search result took into account all search results, even those fragments whose length was only 16 bp (Table 3). The NUMTs found in scaffolds and mitochondrial DNA fragments found in pseudogenes and transcriptome are listed in supplementary materials (Table S1).

In the case of various mtDNA gene fragments, such as *TRNA-MET*, *COX1*, and *ND4* where their number is higher in the transcriptome than in the nuclear genome, it may be the result of the presence of multiple mRNA transcripts or ncRNA forms in the transcriptome. The variability of mRNA and ncRNA forms may be caused by alternative splicing. Moreover, the transcriptome is changing during the lifespan and may depend on the type of tissue [29]. It is not excluded that some NUMT are parts of protein-coding genes and/or participate in gene regulation through ncRNA formation. In this research, the authors focused on describing the presence of mitochondrial DNA in the nuclear genome, while the investigation of the role of NUMTs in gene expression and regulation in *D. magna* should be verified experimentally in further analyses.

NUMTs were used to define characteristics and to clarify phylogenetic inconsistencies suggested by paralog sequences [18,30]. The analysis performed by Mishmar et al. [31] revealed that mtDNA fragments, which were integrated into the nucleus before the radiation of modern human mtDNAs, confirming that mtDNAs similar to today’s African macro-haplogroup L were the first human mtDNAs. The analysis of NUMTs in *D. magna* revealed that the latest evolutionary sequences are pseudogenes derived from the sequence *TRNA-MET* as the homology of the entire gene sequence was 100%. The homology of *TRNA-MET* gene fragments localized in transcriptome is below 100%, however, it could be a result of gene modifications after expression (Table S1). In addition, all mtDNA fragments in transcriptome might differ from each other due to different ways of modulating gene expression depending on the tissue. The lowest homology (70.33%) was characteristic for the pseudogene derived from the mtDNA D-loop sequence located on LG5 (Table S1). It may probably be the oldest element of mitochondrial DNA incorporated into the nuclear genome. However, due to the high degree of mutation in the D-loop, the thesis requires further verification. Similarly, NUMTs derived from *TRNA-ALA* and *TRNA-SER1* may have been one of the first sequences derived from mtDNA. However, by assessing only sequence homology, the sequence of incorporation of mitochondrial pseudogenes into the nuclear genome cannot be determined. Nevertheless, as observed by Mishmar et al. [31], the nuclear genome accumulates mutational changes at a much slower rate than mtDNA. Hence, the sequences of “recent” NUMTs can provide valuable information about the mtDNA sequences of the earliest humans.

NUMTs exhibit different degrees of homology to their mitochondrial counterparts. They are variable in size, evenly distributed within and among chromosomes, and, in some cases, they are highly rearranged and/or fragmented [32]. However, the mitochondrial chromosome’s size does not correlate with the NUMT frequency or size distribution [19]. The transfer to the nucleus can be influenced by mitochondria’s vulnerability to stress and other factors that may cause the escape of mtDNA to the cytoplasm [32]. Mutations in mtDNA may occur in the entire mitochondrial genome; however, they are most frequently detected in the hypervariable regions of D-loops [33,34,35].

The number of mutations as well as their incidence in the D-loop area may be related to the number of NUMTs occurring in the nuclear genome. The higher the mutation rate, the greater the likelihood of transfer of the D-loop fragment into the cytoplasm followed by its incorporation into the nuclear genome as a pseudogene. In the *D. magna* genome, the highest numbers of pseudogenes from the D-loop (147) were observed, and only 23 of them had 100% sequence homology (Table 1).

No pseudogenes derived from the *TRNA-ILE* gene sequence were observed in the nuclear genome (Table 1). However, the detailed search of *TRNA-ILE* fragments in pseudogenes revealed the presence of three of them in LOC116920659, LOC116923942, and LOC116927475 and two fragments in the transcriptome (Table S1). The difference between the number of NUMTs and pseudogenes and transcriptome for the *TRNA-ILE* gene might be the result of the searching method. Pseudogenes were generated by automated computational analysis using gene prediction method: Gnomon [36]. Gnomon is a gene prediction HMM-based program. The core algorithm is based on Genscan which uses a 3-periodic fifth-order Hidden Markov Model (HMM) for the coding propensity score and incorporates descriptions of the basic transcriptional, translational, and splicing signals, as well as length distributions and compositional features of exons, introns, and intergenic regions. For the genes for which no experimental information is available, Gnomon is creating conventional ab initio predictions [36]. As a result of this prediction, the proposed gene contains the fragment of *TRNA-ILE*. The presence of this sequence fragments derived from mtDNA cannot be excluded, however, further experimental analysis of the protein should be held. Due to the lack of comparison with other genome sequences for *D. magna* the authors could not exclude any of the results. The parameters of the Gnomon prediction were widely used in the description of other model organisms’ genomes i.e., *Arabidopsis thaliana*, *Danio rerio*, *Mus musculus*, and *Drosophila melanogaster* [36]. Thus, the authors recognize the possibility of occurrence of tRNA-ILE-derived NUMTs in the *D. magna* genome.

In the human genome, NUMTs are commonly associated with repetitive elements, suggesting a possible role for transposable elements in mtDNA integration in the nuclear genome [31]. Certain NUMTs are repeated multiple times within the human genome [32,37]. In the case of the *D. magna* genome, some NUMTs were also observed, which were repeated many times in different linkage groups (Table S1). However, their association with repetitive elements in the nuclear genome requires additional research. The average levels of NUMT sequence homology for the individual linkage groups do not differ significantly from each other, which may indicate a random and even inclusion of sequence fragments into each of them (Table 4).

The cytochrome C oxidase subunit I (*COI*) has possibly been the most commonly studied marker. However, its popularity is mainly associated with its use as a marker for the DNA barcoding of animal diversity [38]. There are several factors causing inadequacy of mtDNA in general and *COI* individually, such as male-biased gene flow, the selection on any mtDNA nucleotide(s) (as the whole genome is one linkage group), retention of ancestral polymorphism, and introgression following hybridization [39]. Presently, there are huge numbers of *COI* sequences in public databases, and most of them have a limited length, generally close to the length of the barcoding region. It is known that the possibility of the presence of NUMTs in the existing data should not be ignored [40]. Since the success of taxonomic differentiation is positively correlated with the barcode length, the minibarcode length is usually kept above 100 bp. For example, an approximately 250-bp region of 16S rRNA can be successfully amplified from various medicinal preparations and food products. It provides the correct identification of animal species [41,42]. Gene fragments that are often used for species identification in *D. magna* are in the following ranges: *COX1* (19–72 bp), *CYTB* (18–46 bp), 12s rRNA (18–72 bp), and 16s rRNA (19–78 bp); each of them constitutes less than 10% of the length of the entire gene (Table 3). Hence, the NUMT sequences of frequently analyzed genes are generally shorter than the respective mitochondrial sequence; thus, the possibility of NUMT co-amplification should decrease with an increased length of the targeted mitochondrial marker. However, it is worth paying attention to the coverage of NUMTs derived from the *TRNA-ARG* (95%), *TRNA-GLU* (97%), and *TRNA-THR* (95%) genes (Table 3). The transcriptome analysis for *TRNA-ARG* revealed the existence of the NUMT in lncRNA sequence transcript variants of the gene LOC116925827 (Table S1). The presence of this gene fragment in a non-coding sequence may suggest its role in gene regulation. The *TRNA-THR*-derived NUMT was identified in mRNA gene transcript variant LOC116930084. On the other hand, the sequence fragment of *TRNA-GLU* was only partially found in the transcriptome in zinc finger CCHC domain-containing protein 7-like mRNA localized in LG2. Perhaps, in these cases, they are not NUMTs but functional gene fragments taking part in gene modulation. Nonetheless, to confirm it, a detailed analysis of the transcriptome on larger research groups should be held.

NUMTs are highly polymorphic in terms of the sequence, homo/heterozygosis status, and presence/absence at a specific locus [43]. These features facilitate the use of NUMTs as specific population markers, as proposed for the human population by Lang et al. [44] The biological importance of NUMTs may correlate with their location on the chromosome. Depending on the insertion location, NUMTs may perturb the function of the genes [45]. Additionally, de novo integration of NUMT pseudogenes into the nuclear genome has an adverse effect in some cases: promoting various disorders and aging, as observed in humans [46]. Chatre and Ricchetti [47] report that migratory mitochondrial DNA can also impact the replication of the nuclear region in *Saccharomyces cerevisiae*.

## 4. Materials and Methods

The whole sequence of the *Daphnia magna* genome, strain SK and annotation of the nuclear and mitochondrial genome of *D. magna* were obtained from GenBank (the accession numbers for the nuclear and mitochondrial genome are GCA_003990815.1 and NC_026914.1, respectively). Each of the mitochondrial genes were aligned separately to detect plausible NUMTs in the nuclear genome. The presence of mtDNA fragments in defined pseudogenes was estimated by the alignment of mtDNA sequences with the GCF_003990815.1_ASM399081v1_pseudogene_without_product.fna sequence. The presence of mtDNA fragments in the transcriptome was established by the search in GCF_003990815.1_ASM399081v1_rna.fna sequence. The detailed results were presented in Supplementary Table S1. The SK strain genome assembly was released on 02-Jan-2019 and was integrated with the high-density genetic linkage map of the strain Xinb3. The presence of NUMTs in the *D. magna* nuclear genome GCA_003990815.1 was evaluated using the BLAST (BLASTN 2.6.0) (Table S1) [48] program implemented within the CLC Genomics Workbench 12.0 software package (https://www.qiagenbioinformatics.com). In the presented study, we implemented a low complexity filter to mask off the query sequence segments with low compositional complexity. Such filtering was used to eliminate statistically significant but biologically uninteresting reports from the BLAST output, leaving the more biologically interesting regions of the query sequence available for specific matching against database sequences. The expectation value (e-value = 10) describing the threshold for reporting matches against database sequences based on the assumption that ten matches are expected to be found merely by chance according to the stochastic model of Karlin and Altschul (1990) [49]. If the E-value ascribed to a match was greater than the expected threshold, no match was reported. The lower value of the threshold caused more stringent searching criteria, leading to fewer chance matches being reported. Higher threshold results in more matches being reported, but many may just match by chance, not due to any biological similarity. The value of match/mismatch, which assigned a score for aligning and evaluated the quality of a pairwise sequence alignment was set to 2/3. The penalty to open gap and penalty to extend gap (Gap Cost.) was set to 5 for existence and 2 for extension. The maximum number of database sequences, where BLAST found matches to a query sequence, to be included in the BLAST report was set to 100.5.

## 5. Conclusions

This article described the first occurrence of mitochondrial pseudogenic sequences (NUMTs) in the nuclear genome of *D. magna*. There was no full sequence homology for two genes: *TRNA-SER1* as well as *TRNA-ALA* NUMTs. The total length of NUMTs in the nuclear genome was 44.391 bp (from 16 to 182 bp), which accounted for 0.042% of the entire genome. The best-matched NUMTs covering more than 90% of the mtDNA gene sequence were identified for the *TRNA-ARG* (95%), and *TRNA-THR* (95%) genes, and they may be included in the functional nuclear RNA molecules. The NUMT length varied from 16 to 63 bp for tRNA genes, from 17 to 108 bp for coding genes, from 18 to 78 bp for rRNA genes, and from 17 to 182 bp for the D–loop region. Therefore, using the product of total DNA isolation in mtDNA studies, co-amplification of nDNA fragments is unlikely especially in the case of amplification of the whole tRNA genes and fragments of other genes the D-loop with a length exceeding 200 bp. It was observed that fragments *TRNA-MET* (from 16 to 18 bp length) had the highest level of sequence homology, which means that they could be evolutionarily the youngest. The lowest degree of homology was found in the pseudogene derived from the mtDNA D-loop sequence. It may probably be the oldest element of mitochondrial DNA incorporated into the nuclear genome; however, due to the high degree of mutation in the D-loop, the thesis requires further analysis and elucidation.

## Figures and Tables

**Table 1:** Percentage content of NUMTs in the linkage groups of the nuclear genome.

Linkage Group (LG)	Physical Length (bp) from Lee et al., 2019	Sum of NUMTs Lengths (bp)	Percentage Content of NUMTs on LG
LG1	14,058,888	5.063	0.036%
LG2	16,351,056	6.071	0.037%
LG3	11,081,246	4.389	0.040%
LG4	10,002,879	4.147	0.041%
LG5	10,116,075	4.600	0.045%
LG6	9,588,688	3.997	0.042%
LG7	10,149,764	4.829	0.048%
LG8	9,006,911	3.533	0.039%
LG9	8,299,553	3.966	0.048%
LG10	8,061,327	3.796	0.047%
Total	106,716,387	44.391	0.042%

**Table 2:** Distribution of mtDNA gene fragments in the linkage groups and the sum of fragment length.

mtDNA Sequence	LG										Number of NUMTs (100% Identical) *	Sum of Gene Fragments (bp)
	1	2	3	4	5	6	7	8	9	10		
*TRNA-GLN*	3	6	5	7	2	5	3	2	5	4	**42** (15)	966
*TRNA-MET*	2	2	1	1	-	-	-	-	-	-	**6** (6)	102
*ND2*	12	18	15	12	12	9	12	12	6	7	**115** (15)	3235
*TRNA-TRP*	3	2	3	3	2	-	-	3	1	2	**19** (6)	447
*TRNA-CYS*	11	13	9	14	5	14	11	10	11	12	**110** (12)	2596
*TRNA-TYR*	-	1	-	-	-	-	-	1	1	1	**4** (1)	100
*COX1*	1	1	2	2	5	3	7	3	3	4	**31** (2)	1175
*TRNA-LEU1*	5	1	9	3	1	6	5	3	1	2	**36** (10)	786
*COX2*	2	6	3	4	14	7	5	5	2	4	**52** (9)	1492
*TRNA-LYS*	-	2	3	-	2	-	2	2	-	1	**12** (3)	290
*TRNA-ASP*	3	-	1	2	-	2	1	3	-	-	**12** (1)	273
*ATP8*	-	5	1	2	-	1	3	2	1	-	**15** (3)	438
*ATP6*	4	7	4	4	5	2	2	1	2	3	**34** (2)	957
*COX3*	3	2	2	-	4	2	3	-	3	4	**23** (3)	717
*TRNA-GLY*	2	3	4	-	2	1	5	2	2	2	**23** (3)	512
*ND3*	17	17	14	6	9	14	10	9	15	2	**113** (21)	2876
*TRNA-ALA*	1	6	1	5	1	1	1	2	-	1	**19** (0)	407
*TRNA-ARG*	2	1	5	5	2	5	-	1	1	3	**25** (4)	695
*TRNA-ASN*	1	1	-	1	-	-	-	-	1	-	**4** (1)	112
*TRNA-SER1*	-	-	-	-	-	-	-	2	-	-	**2** (0)	52
*TRNA-GLU*	5	7	4	2	4	3	4	4	5	-	**38** (8)	924
*TRNA-PHE*	-	1	-	-	2	1	-	2	2	1	**9** (2)	187
*ND5*	17	15	7	11	7	10	10	3	10	8	**98** (10)	3244
*TRNA-HIS*	-	3	1	2	2	3	7	1	3	2	**24** (6)	536
*ND4*	7	4	3	4	2	1	2	4	3	7	**37** (1)	1194
*ND4L*	4	3	3	1	1	2	1	2	-	6	**23** (2)	615
*TRNA-THR*	5	2	-	-	2	-	3	3	2	-	**17** (9)	355
*TRNA-PRO*	-	-	1	3	-	-	1	-	1	-	**6** (1)	151
*ND6*	9	14	12	7	5	8	14	10	4	8	**91** (7)	2445
*CYTB*	8	14	7	5	6	5	4	3	6	4	**62** (8)	1631
*TRNA-SER2*	9	7	9	3	9	6	5	4	5	11	**68** (16)	2644
*ND1*	15	12	9	13	6	11	13	6	5	6	**96** (16)	1486
*TRNA-LEU2*	-	3	-	-	1	1	-	1	-	-	**6** (2)	135
*16S rRNA*	11	19	3	7	15	8	11	11	10	10	**105** (10)	3700
*TRNA-VAL*	2	2	1	1	3	1	2	2	-	-	**14** (4)	320
*12S rRNA*	12	16	6	7	5	6	12	5	13	10	**92** (10)	2617
*TRNA-ILE*	-	-	-	-	-	-	-	-	-	-	**0**	0
*D-LOOP*	13	12	22	13	29	12	12	10	11	13	**147** (23)	3979
***SUM***	**189**	**228**	**170**	**150**	**165**	**150**	**171**	**134**	**135**	**138**	**1630** (253)	
**Sum of fragments in the LG (bp)**	5063	6071	4389	4147	4600	3997	4829	3533	3966	3796		44.391

* The counts of NUMTs that were 100% identical with the sequence from mtDNA are indicated in brackets.

**Table 3:** Minimal and maximal length of each mtDNA gene fragments located in the nuclear genome.

Sequence	Length (in bp)	Min (in bp)	% Min	Max (in bp)	% Max
***TRNA-GLN***	68	16	24%	44	65%
***TRNA-MET***	65	16	25%	18	28%
***ND2***	987	18	2%	58	6%
***TRNA-TRP***	64	16	25%	38	59%
***TRNA-CYS***	64	16	25%	40	63%
***TRNA-TYR***	64	18	28%	35	55%
***COX1***	1537	19	1%	72	5%
***TRNA-LEU1***	68	16	24%	39	57%
***COX2***	679	18	3%	52	8%
***TRNA-LYS***	70	17	24%	35	50%
***TRNA-ASP***	63	17	27%	32	51%
***ATP8***	168	17	10%	54	32%
***ATP6***	675	18	3%	47	7%
***COX3***	789	19	2%	94	12%
***TRNA-GLY***	63	16	25%	32	51%
***ND3***	354	17	5%	99	28%
***TRNA-ALA***	62	19	31%	28	45%
***TRNA-ARG***	64	16	25%	**61**	**95%**
***TRNA-ASN***	67	16	24%	45	67%
***TRNA-SER1***	65	25	38%	27	42%
***TRNA-GLU***	65	16	25%	**63**	**97%**
***TRNA-PHE***	68	16	24%	26	38%
***ND5***	1708	19	1%	94	6%
***TRNA-HIS***	63	16	25%	42	67%
***ND4***	1315	19	1%	108	8%
***ND4L***	306	17	6%	38	12%
***TRNA-THR***	63	16	25%	**60**	**95%**
***TRNA-PRO***	64	16	25%	34	53%
***ND6***	504	18	4%	64	13%
***CYTB***	1133	18	2%	46	4%
***ND1***	927	18	2%	88	9%
***TRNA-SER2***	69	16	23%	35	51%
***TRNA-LEU2***	67	16	24%	27	40%
***16S RRNA***	1373	19	1%	78	6%
***TRNA-VAL***	72	16	22%	38	53%
***12S RRNA***	752	18	2%	72	10%
***TRNA-ILE***	64	-	-	-	-
**D-LOOP**	289	17	6%	**182**	**63%**

**Table 4:** Mean % identity of mtDNA gene fragments located in the linkage groups.

Sequence	LG1	LG2	LG3	LG4	LG5	LG6	LG7	LG8	LG9	LG10	Mean % Identity–Gene
***TRNA-GLN***	93.8	88.8	94.3	92.9	92.3	85.1	97.1	89.4	90.5	95.0	**91.6**
***TRNA-MET***	**100.0**	**100.0**	**100.0**	**100.0**	-	-	-	-	-	-	**100.0**
***ND2***	88.7	91.4	90.2	88.0	89.3	88.4	91.2	90.0	87.2	87.2	**89.5**
***TRNA-TRP***	90.9	95.2	88.3	90.2	93.8	-	-	89.7	**100.0**	94.0	**91.8**
***TRNA-CYS***	92.6	89.7	89.1	89.9	88.4	93.3	91.4	87.6	88.7	92.4	**90.5**
***TRNA-TYR***	-	85.7	-	-	-	-	-	**100.0**	94.7	97.1	**94.4**
***COX1***	79.5	92.3	82.6	94.6	87.8	82.2	88.3	90.7	92.8	95.4	**89.1**
***TRNA-LEU1***	91.0	**100.0**	92.5	91.9	**100.0**	93.6	91.5	95.2	86.2	83.3	**92.2**
***COX2***	81.6	95.4	86.5	87.5	92.1	90.2	91.1	89.0	85.0	89.0	**90.2**
***TRNA-LYS***	-	95.2	86.5	-	98.6	-	95.1	91.9	-	90.5	**92.6**
***TRNA-ASP***	96.8	-	95.0	84.4	-	85.6	90.9	91.5	-	-	**90.9**
***ATP8***	-	88.4	88.5	84.8	-	**100.0**	84.5	87.7	90.9	-	**88.0**
***ATP6***	86.9	85.1	90.4	88.4	93.1	90.3	86.9	95.2	85.5	92.7	**88.9**
***COX3***	84.9	90.5	87.6	-	90.5	91.3	97.1	-	88.3	94.6	**90.9**
***TRNA-GLY***	90.4	92.1	92.0	-	95.2	90.9	94.3	85.0	95.2	88.3	**92.0**
***ND3***	90.2	91.5	89.2	88.5	96.1	91.1	90.3	89.9	91.5	95.7	**91.0**
***TRNA-ALA***	90.5	93.7	94.7	89.8	94.7	90.9	88.0	90.7	-	94.7	**91.9**
***TRNA-ARG***	89.0	90.5	92.0	94.3	89.7	87.0	-	90.9	94.7	97.7	**91.7**
***TRNA-ASN***	84.6	**100.0**	-	88.0	-	-	-	-	95.6	-	**92.0**
***TRNA-SER1***	-	-	-	-	-	-	-	90.3	-	-	**90.3**
***TRNA-GLU***	89.8	90.8	89.3	90.2	93.0	95.1	87.6	91.6	96.2	-	**91.5**
***TRNA-PHE***	-	**100.0**	-	-	90.1	90.5	-	95.5	92.6	91.3	**93.1**
***ND5***	89.5	88.0	86.1	92.5	89.4	92.4	86.8	86.0	89.5	88.6	**89.2**
***TRNA-HIS***	-	95.1	**100.0**	**100.0**	80.7	87.6	91.2	88.9	89.0	**100.0**	**91.8**
***ND4***	89.5	86.1	90.9	87.3	93.0	89.7	87.3	88.5	88.7	94.1	**89.8**
***ND4L***	86.1	87.4	84.6	82.9	**100.0**	88.9	95.0	87.3	-	90.8	**88.5**
***TRNA-THR***	98.3	89.5	-	-	95.0	-	98.3	**100.0**	94.0	-	**96.6**
***TRNA-PRO***	-	-	91.3	90.7	-	-	**100.0**	-	92.6	-	**92.7**
***ND6***	92.9	89.0	89.7	92.6	87.8	90.9	89.1	89.2	88.7	92.6	**90.2**
***CYTB***	91.1	89.4	89.8	93.0	91.1	87.1	88.9	93.4	92.2	90.9	**90.5**
***ND1***	90.7	92.7	93.1	88.3	92.3	88.3	91.5	92.5	90.6	86.1	**90.6**
***TRNA-SER2***	95.3	92.3	94.4	95.2	89.4	93.6	96.6	90.2	90.3	88.8	**92.3**
***TRNA-LEU2***	-	92.0	-	-	85.2	85.2	-	**100.0**	-	-	**91.1**
***16S RRNA***	86.3	87.6	81.7	91.9	87.1	88.4	86.4	87.9	86.1	92.7	**87.8**
***TRNA-VAL***	**100.0**	83.5	90.5	92.0	90.9	90.5	83.3	**100.0**	-	-	**91.4**
***12S RRNA***	89.1	89.9	88.2	87.8	90.2	88.9	89.2	93.0	93.1	85.5	**89.5**
**D-LOOP**	91.9	90.0	91.7	91.8	88.8	91.0	90.3	92.6	91.5	88.0	**90.6**
**Mean % Identity–LG**	**90.6**	**90.3**	**90.3**	**90.5**	**90.4**	**90.2**	**90.4**	**90.6**	**90.6**	**90.7**	**90.4**

**Table 5:** The comparison of the number and length of mitochondrial DNA fragments found in the nuclear genome (NUMTs), pseudogenes, and the transcriptome.

	NUMTs		mtDNA Fragments Found in Pseudogenes		mtDNA Fragments Found in Transcriptome	
Gene	Number	Greatest Length (bp)	Number	Greatest Length (bp)	Number	Greatest Length (bp)
*TRNA-GLN*	46	44	13	19	23	35
*TRNA-MET*	6	18	5	18	21	29
*ND2*	128	58	47	39	36	58
*TRNA-TRP*	23	38	11	30	58	38
*TRNA-CYS*	136	40	23	26	101	34
*TRNA-TYR*	4	35	0	0	5	25
*COX1*	37	72	11	72	52	61
*TRNA-LEU1*	38	39	5	20	28	23
*COX2*	70	53	21	38	14	39
*TRNA-LYS*	14	35	8	35	7	31
*TRNA-ASP*	14	32	3	29	18	39
*ATP8*	18	54	2	15	10	26
*ATP6*	38	64	11	24	6	64
*COX3*	29	94	15	27	25	28
*TRNA-GLY*	23	32	13	37	31	48
*ND3*	131	99	28	99	27	53
*TRNA-ALA*	21	28	5	18	57	28
*TRNA-ARG*	29	61	18	31	85	61
*TRNA-ASN*	4	45	4	19	3	45
*TRNA-SER1*	3	27	0	0	1	20
*TRNA-GLU*	46	63	15	29	27	30
*TRNA-PHE*	10	26	5	24	1	26
*ND5*	107	94	19	65	70	43
*TRNA-HIS*	26	42	7	21	28	27
*ND4*	46	108	25	33	73	33
*ND4L*	25	38	10	38	5	30
*TRNA-THR*	18	60	9	22	46	60
*TRNA-PRO*	6	34	1	13	19	38
*ND6*	106	64	21	41	97	42
*CYTB*	71	59	27	38	19	35
*TRNA-SER2*	74	35	29	37	33	31
*ND1*	107	88	45	88	24	34
*TRNA-LEU2*	7	27	10	23	9	27
*16S rRNA*	123	78	63	59	100	46
*TRNA-VAL*	19	38	9	20	8	32
*12S rRNA*	136	72	10	25	42	51
*TRNA-ILE*	0	0	3	20	2	20
D-LOOP	170	182	48	34	64	50
Sum	1909		599		1275	

## Supplementary Materials

Supplementary materials can be found at https://www.mdpi.com/1422-0067/21/22/8725/s1. Table S1: The presence and evaluation of all NUMTs found in the BLAST tool as well as mtDNA fragments found in pseudogenes and transcriptome.

## Abbreviations

NUMTs	nuclear copies of mitochondrial dna
BP	base pairs
LG	linkage groups
KIT	Korea Institute of Toxicology
EST	expressed sequence tags
nDNA	nuclear DNA
mtDNA	mitochondrial DNA
BLAST	Basic Local Alignment Search Tool
ND1-6 and ND4L	NADH dehydrogenase subunits 1–6 and subunit 4L
CO1-3 or COX1-3	cytochrome oxidase subunits I-III
ATP6 and 8	ATPase subunit 6 and 8
CYTB	cytochrome b
12S rRNA	gene for small subunit ribosomal RNA
16S rRNA	gene for large subunit ribosomal RNA
TRNA	gene coding transfer RNA
